# Homodimerization of the Lymph Vessel Endothelial Receptor LYVE-1 through a Redox-labile Disulfide Is Critical for Hyaluronan Binding in Lymphatic Endothelium*[Fn FN2]

**DOI:** 10.1074/jbc.M116.736926

**Published:** 2016-10-12

**Authors:** Suneale Banerji, William Lawrance, Clive Metcalfe, David C. Briggs, Akira Yamauchi, Omer Dushek, P. Anton van der Merwe, Anthony J. Day, David G. Jackson

**Affiliations:** From the ‡ Medical Research Council Human Immunology Unit, Weatherall Institute of Molecular Medicine, John Radcliffe Hospital, Headington, Oxford OX3 9DS, United Kingdom,; § Sir William Dunn School of Pathology, University of Oxford, South Parks Road, Oxford OX1 3RE, United Kingdom,; ‖ Department of Biochemistry, Kawasaki Medical School, 577 Matsushima, Kurashiki 701-0192, Japan, and; ¶ Wellcome Trust Centre for Cell Matrix Research, Faculty of Biology, Medicine, and Health, University of Manchester, Oxford Road, Manchester M13 9PT, United Kingdom

**Keywords:** disulfide, endothelium, hyaluronan, receptor, redox regulation, LYVE-1, avidity, dimer, labile, lymphatic

## Abstract

The lymphatic vessel endothelial receptor LYVE-1 is implicated in the uptake of hyaluronan (HA) and trafficking of leukocytes to draining lymph nodes. Yet LYVE-1 has only weak affinity for hyaluronan and depends on receptor clustering and higher order ligand organization for durable binding in lymphatic endothelium. An unusual feature of LYVE-1 not found in other HA receptors is the potential to form disulfide-linked homodimers. However, their influence on function has not been investigated. Here we show LYVE-1 homodimers are the predominant configuration in lymphatic endothelium *in vitro* and *in vivo*, and formation solely requires the unpaired cysteine residue Cys-201 within the membrane-proximal domain, yielding a 15-fold higher HA binding affinity and an ∼67-fold slower off-rate than the monomer. Moreover, we show non-dimerizing LYVE-1 mutants fail to bind HA even when expressed at high densities in lymphatic endothelial cells or artificially cross-linked with antibody. Consistent with these findings, small angle X-ray scattering (SAXS) indicates the Cys-201 interchain disulfide forms a hinge that maintains the homodimer in an “open scissors” conformation, likely allowing arrangement of the two HA binding domains for mutual engagement with ligand. Finally, we demonstrate the Cys-201 interchain disulfide is highly labile, and selective reduction with TCEP-HCl disrupts LYVE-1 homodimers, ablating HA binding. These findings reveal binding is dependent not just on clustering but also on the biochemical properties of LYVE-1 homodimers. They also mark LYVE-1 as the first Link protein superfamily member requiring covalent homodimerization for function and suggest the interchain disulfide acts as a redox switch *in vivo*.

## Introduction

Initial lymphatic vessels are composed largely of specialized endothelial cells that are adapted for the uptake of fluid, cells, and macromolecules from the interstitium and their transport to draining lymph nodes (1–3). LYVE-1, the primary receptor for hyaluronan (HA)^2^ in lymphatic endothelium is a well known and widely utilized marker for these vessels, but its precise physiological function has remained largely enigmatic (4–6). The protein was originally identified and characterized as a homologue of CD44, the primary HA receptor in leukocytes, and as a new member of the Link module superfamily of C-type lectins (7) to which most known HA-binding proteins belong (4, 8, 9). Similar to CD44 in terms of predicted structure, LYVE-1 has a single N-terminal HA binding domain that comprises a consensus Link module flanked by N- and C-terminal extensions analogous to those that regulate ligand binding in the former receptor (9–12). Although this domain contains an HA binding surface of similar dimensions to that of CD44, the HA interaction is markedly distinct insofar as it is likely dominated by charged rather than hydrophobic residues and exhibits a weaker binding affinity that results in dissociation even at relatively low ionic strength (10).

In common with CD44, recombinant LYVE-1 in some transfected cell types binds HA efficiently, whereas in others binding is suppressed due in part to receptor sialylation that appears to block the interaction with ligand (5, 10). Most notably, LYVE-1 in native lymphatic endothelium displays little if any detectable binding to high molecular weight (HMW) HA, a seemingly paradoxical feature that initially suggested the receptor might be largely inactive *in vivo*. Just recently, however, we reported that native LYVE-1 is fully functional in this respect, although only when expressed above a critical surface threshold density or grouped in discrete surface microclusters (13). In a similar manner, LYVE-1 was shown to bind HA efficiently when the sugar is organized as cross-linked complexes with the hyaladherin TSG-6 or when it is arranged either on migrating leukocytes or in the dense surface capsule of pathogenic group A streptococci that exploit the receptor for lymphatic dissemination (13, 14). These various findings have reinforced the notion that LYVE-1 is a tightly regulated and physiologically important receptor and that its inherently low affinity for HA confers a dependence on multivalent interactions for durable binding through avidity (15).

One intriguing feature of LYVE-1 that has received little attention but which has potentially important consequences for HA binding is its potential to form disulfide-linked homodimers. Unlike the other major Link superfamily HA receptors CD44 and the liver sinusoidal receptor known as Stabilin-2 or HARE (16, 17), LYVE-1 contains a single highly conserved unpaired cysteine within the glycosylated membrane-proximal domain that is predicted to form an interchain disulfide bridge (4, 18). Indeed, this feature delineates the receptor from all other Link module superfamily members, which function as single chain HA-binding proteins, albeit in some cases composed of tandemly repeated Link modules (8).

Here we have evaluated the capacity of LYVE-1 to form homodimers in native lymphatic endothelium and investigated the significance of dimerization in terms of its consequences for HA binding affinity and receptor function in this context. We identify Cys-201 as the critical cysteine residue generating the interchain disulfide and show by means of Biacore analysis that homodimer formation compensates for the inherently weak affinity of the LYVE-1 monomer and enhances HA binding some ×15-fold. Moreover, we reveal that disruption of the Cys-201 disulfide bridge in the homodimer by site-directed mutagenesis ablates HA binding in primary lymphatic endothelial cells and that binding cannot be reconstituted by the monomer even at the highest levels of surface expression or by artificial cross-linking with LYVE-1 antibody. In addition, we present structure-based evidence from small angle X-ray scattering (SAXS) of LYVE-1 ectodomains that the Cys-201 intermolecular disulfide forms a hinge that maintains the homodimer in a splayed open scissors conformation that may well orient the two monomer HA binding domains for optimal engagement with ligand. Lastly, we present evidence that the Cys-201 interchain disulfide within the native LYVE-1 homodimer is highly labile, suggesting that dimerization and hence HA binding may be influenced by the extracellular redox microenvironment. These findings uncover additional, unexpected layers of complexity in the regulation of LYVE-1 HA interactions and attest to their functional importance in supporting HA-mediated interactions in the lymphatic compartment.

## Results

### 

#### 

##### LYVE-1 Is Expressed Predominantly as Homodimers in Primary Lymphatic Endothelium

**FIGURE 1.**
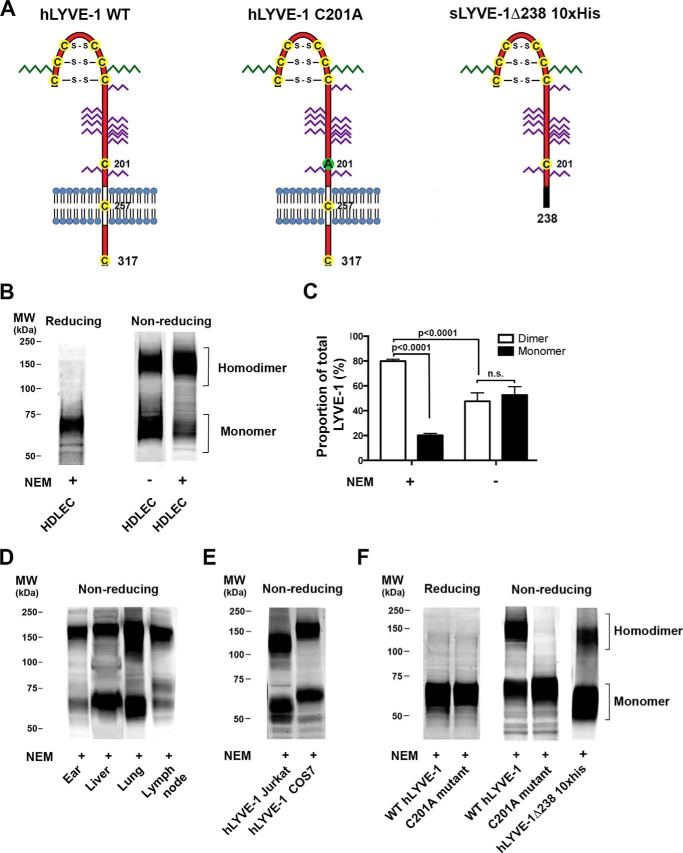
**LYVE-1 in lymphatic endothelium is organized largely as homodimers linked via the critical disulfide Cys-201.** The self-association status of LYVE-1 in native HDLEC and transfected cells and the potential involvement of Cys-201 were assessed using non-reducing SDS-PAGE and Western blotting. *A*, schematic representations of wild-type hLYVE-1 and hLYVE-1 C201A in which the unpaired cysteine 201 in the membrane proximal domain predicted to form an intermolecular disulfide was mutated to alanine; also shown is the soluble full ectodomain construct hLYVE-1 Δ238 His_10_. Positions of the three disulfides in the extended Link module and two further unpaired cysteines in the transmembrane and cytoplasmic domains are indicated. *N*-Linked sugars are shown in *green*, and *O*-linked sugars are in *purple. B*, reducing and non-reducing SDS-PAGE/Western blots of LYVE-1 in primary HDLEC showing predominant organization as the homodimer and its disassembly in HDLEC lysed in the absence of NEM as indicated. *C*, *bar chart* showing quantitative comparison of monomer and dimer band intensities from equivalent HDLEC Western blots (mean ± S.E. *n* = 4) as determined by densitometric scanning. Statistical *p* values were obtained using the two-tailed unpaired *t* test (*n.s.*, non-significant). *D* and *E*, non-reducing SDS-PAGE/Western blots of LYVE-1 in different mouse tissues and transfected COS7 and Jurkat T cells lysed in the presence of NEM as indicated. *F*, reducing and non-reducing SDS-PAGE/Western blots of 293T cells transfected with either full-length wild-type LYVE-1 or C201A mutants and of CHO cell-derived soluble hLYVE-1 Δ238 His_10_ protein showing the critical involvement of Cys-201 in homodimer formation. Samples were prepared in the presence of NEM as indicated. All blots were probed with affinity-purified polyclonal goat anti-human or mouse LYVE-1 antibodies followed by anti-goat IRdye® 800 before imaging with a Licor® Odyssey scanner. All data are from representative experiments that were repeated at least three times.

The original cloning and sequencing of hLYVE-1 cDNA revealed a single unpaired cysteine (Cys-201) near the base of the *O*-glycosylated membrane stalk region that is conserved in all vertebrate sequences with the exception of fish and Amphibia (4, 18), indicating the potential for LYVE-1 to form homodimers through intermolecular disulfide bonding (see Fig. 1*A*). However, no studies to date have addressed the biochemical properties of LYVE-1 homodimers, their expression in normal tissue, or their significance for receptor function.

To characterize disulfide-linked homodimer formation by native LYVE-1, we first compared the mobility of the receptor in whole cell lysates of primary human dermal endothelial cells (HDLEC) subjected to SDS-PAGE under reducing and non-reducing conditions followed by Western blotting with polyclonal LYVE-1 antibody. Under non-reducing conditions, shown in Fig. 1, *B* and *C*, the receptor migrated as two broad bands of almost equal intensity with apparent molecular masses ranging between 60–80 and 120–180 kDa, corresponding to mixed glycoforms of the monomer and its disulfide-linked homodimer, whereas only the 60–80-kDa receptor was present under reducing conditions with 2-mercaptoethanol. Significantly, however, when the thiol-modifying agent *N*-ethylmaleimide (NEM) was included during HDLEC lysis the majority of the receptor (80% as determined by quantitative densitometry) migrated as the 120–180-kDa dimer form (Fig. 1, *B* and *C*). This result suggests that dermal LEC synthesize predominantly LYVE-1 homodimers, but these are highly susceptible to reduction by endogenous thiol-based antioxidants released during cell lysis. A similar preponderance of LYVE-1 homodimers (80–90%) was detected in samples of freshly resected mouse ear skin and lymph node (Fig. 1*D*), confirming they also represent the dominant form of the receptor in lymphatic endothelium *in vivo*. In contrast, mouse liver and lung tissue in which LYVE-1 is expressed mostly in hepatic sinusoids and pulmonary blood vessels (4, 11) contained heterogeneous (∼2:1) mixtures of homodimer and monomer. This was also the case for LYVE-1 in transfected Jurkat and COS7 cells despite the inclusion of NEM (Fig. 1*E*). The variation in apparent molecular weight ranges of both monomer and homodimer between these different cell and tissue types reflects the influence of lineage-specific glycosylation patterns, as outlined previously (19). These results reveal that LYVE-1 is expressed preferentially as homodimers in native human and mouse lymphatic endothelium but that the dimer:monomer ratio can vary between cell types and tissues, likely as a result of diverse redox environments and innate cell-based differences in biosynthesis.

##### The Critical Disulfide Is Generated by Cys-201 in the Membrane Proximal Domain

Next, to formally identify the residue constituting the LYVE-1 intermolecular disulfide as either Cys-201 or one of the two additional conserved residues Cys-257 or Cys-317 present in the transmembrane anchor and cytoplasmic tail respectively, we constructed a hLYVE-1 mutant, C201A, converting cysteine to alanine by site-directed mutagenesis. This was then compared with wild-type hLYVE-1 for its ability to form covalent dimers in transfected HEK 293T cells as assessed by SDS-PAGE/Western blotting. The results (Fig. 1*F*) showed the wild-type receptor formed mostly 120–160-kDa dimers as expected, (2:1 dimer:monomer ratio), whereas the C201A mutant formed no dimers and was exclusively monomeric. Furthermore, a soluble hLYVE-1 ectodomain construct encompassing Cys-201 (hLYVE-1 Δ238 His_10_) but lacking the transmembrane domain and cytoplasmic tail formed similar dimer:monomer mixtures indicating these components are not required to form the intermolecular disulfide.

##### LYVE-1 Homodimerization Exerts a Large Increase in HA Binding Affinity

Previously, we showed that LYVE-1 extracellular domains expressed as immunoglobulin Fc fusion proteins display only low to intermediate binding affinity for HA when estimated by either conventional plate binding assays or surface plasmon resonance (10). However, an important caveat of this expression method is that the incorporated Fc tag forces artificial dimerization via the constituent disulfide bridge in the Ig hinge region, a factor that likely influences measured binding affinities through avidity effects. Furthermore, the LYVE-1 partners within these IgFc fusion proteins comprise a mixture of unlinked and disulfide-linked species. Hence, to allow differentiation of the two species we employed an alternative strategy of expressing mature LYVE-1 ectodomains as soluble proteins fused to a C-terminal His_10_ tag using a glutamine synthase-based amplification system in Chinese hamster ovary (CHO) cells, a background that elicits partial but incomplete sialylation of LYVE-1 (19) and is, therefore, compatible with these HA binding studies.

**FIGURE 2.**
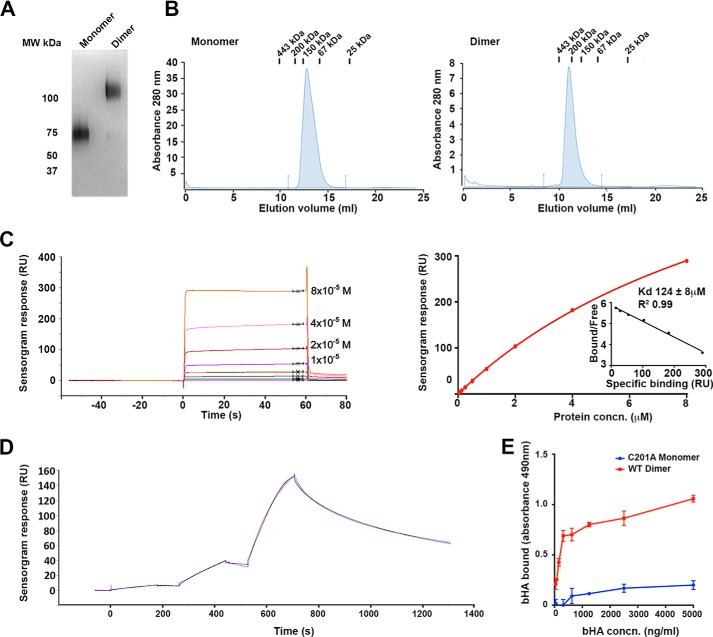
**LYVE-1 homodimerization greatly increase HA binding affinity.** Binding affinities of soluble hLYVE-1 Δ238 monomer (C201A non-dimerizing mutant) and homodimer derived from transfected CHO cells (*A* and *B*) were determined by surface plasmon resonance analysis with HMW HA immobilized on the sensor chip (*C* and *D*) and by microtiter plate analysis (*E*) with immobilized receptor and bHA. *A*, SDS-PAGE analysis of the purified hLYVE-1 monomer and dimer under non-reducing conditions, detected by staining with Coomassie Blue. *B*, Superdex-200 size exclusion chromatography profiles of purified monomer and dimer preparations with elution positions of molecular weight calibration markers shown at *top. C*, Biacore sensorgram and the associated binding curve with Scatchard plot (*inset*; *RU*, response units) for hLYVE-1 monomer measured at equilibrium with varying analyte concentrations (0.5–128 μm). The *K_D_* value was determined from independent replicate analyses (mean ± S.E. *n* = 3) by fitting the data to a 1:1 Langmuir binding isotherm. The Scatchard plot indicates this binding model fits the data well. *D*, Biacore sensorgram for hLYVE-1 homodimer measured as a single-cycle kinetic experiment using sequential injections at LYVE-1 concentrations of 12.8, 64, and 320 nm (*purple line*). Evaluation and fitting was performed using Biaevaluation software assuming bivalency for the analyte (*black line*). Further analysis including rationalization of the number of parameters is included in the supplemental information. *E*, analysis of bHA binding to hLYVE-1 Δ238 monomer (non-dimerizing C201A mutant) or intact LYVE-1 Δ238 homodimer immobilized on a microtiter plate, detected colorimetrically with streptavidin HRP (absorbance 490 nm) as described under “Experimental Procedures.” Values are the mean ± S.E. *n* = 3. Data shown are from representative experiments that were repeated at least three times.

As expected, the LYVE-1 proteins recovered from CHO cell supernatants contained a mixed population of monomers and disulfide-linked dimers (see Fig. 1) that was then subjected to multiple rounds of size exclusion chromatography to separate the two species. For our initial studies the resulting LYVE-1 monomer preparation was subjected to alkylation with *N*-ethylmaleimide to inhibit post-separation dimer formation. However, in subsequent studies we expressed LYVE-1 as the non-dimerizing C201A mutant to avoid the potential off-target effects of exposure to the thiol modification reagent. As shown in Fig. 2*A*, the isolated LYVE-1 homodimers and C201A monomers migrated as single bands with approximate molecular masses in the range of 120 and 60–80 kDa, respectively, on SDS-PAGE. In addition, the purity of the preparations was confirmed by size exclusion chromatography, which yielded a single major peak in each case (Fig. 2*B*). Notably, the homodimer and monomer peaks eluted at similar positions to the 200- and 150-kDa calibration markers, respectively, indicating larger hydrodynamic volumes than would have been anticipated from the predicted molecular mass, most likely due to the influence of the highly glycosylated stalk-like juxtamembrane domain (19). These purified proteins served as analytes for HMW (end-biotinylated) HA immobilized on a streptavidin-coated Biacore sensor chip.

As shown in Fig. 2*C*, the non-dimerizing C201A monomer bound to HA with fast kinetics, reaching equilibrium within 5s. This enabled the affinity to be measured directly, yielding a mean *K_D_* value of 124 ± 8 μm by equilibrium binding analysis, although the binding kinetics were too fast for accurate measurement of individual rate constants. In contrast, the LYVE-1 homodimer bound HA with much slower kinetics (Fig. 2*D*), failing to reach equilibrium even after 10 min and necessitating the use of kinetic measurements for estimation of apparent *K_D_*.

Accordingly, the bivalent analyte model (provided by the Biacore T200 evaluation software) was fitted to the experimental data, providing estimates for the first step solution on-rate (*k*^1^_on_ =0.0692 μm^−1^s^−1^), the second step on-rate (*k*^2^_on_ = 7.46 μm^−1^s^−1^), and the monovalent off-rate (*k*_off_ = 8.55 s^−1^) Next, we derived an expression for the apparent dissociation constant representing half-maximal surface ligand occupancy (see the supplemental information), which yielded a *K_D_* = 8.2 μm, indicating that binding of the dimer was some 15-fold “tighter” compared to the monomer on a per molecule basis. We also derived an expression for the effective off-rate of bivalent LYVE-1 to obtain a k*off value of 0.128 s^−1^, which is 67-fold slower than the monovalent off-rate. The magnitude of this difference indicates that even a small change in the proportion of LYVE-1 dimer in LECs, elicited *e.g.* by an alteration in extracellular redox potential, could shift the balance between transient and more stable binding of appropriate HA complexes or HA encapsulated pathogens *in vivo*. Moreover, the ∼×100-fold faster second step on-rate (*k*^2^_on_) for HA binding compared with *k*^1^_on_ might suggest some cooperativity between the two constituent HA binding units in the homodimer.

To further validate the binding affinities for LYVE-1 monomers and homodimers obtained from the above Biacore analyses, we performed analogous binding studies in the reverse orientation by immobilizing fixed quantities of either LYVE-1 monomer or homodimer in wells of a microtiter plate and applying increasing concentrations of soluble HMW biotinylated HA (bHA) for subsequent detection using streptavidin. As shown in Fig. 2*E*, the amount of HA bound by the homodimer was much greater than that bound by monomer for all concentrations of HA tested, further substantiating the finding of a higher binding affinity for the LYVE-1 homodimer in the Biacore studies.

##### Low Resolution Solution Structure of the LYVE-1 Homodimer and Significance for Biological Function

**FIGURE 3.**
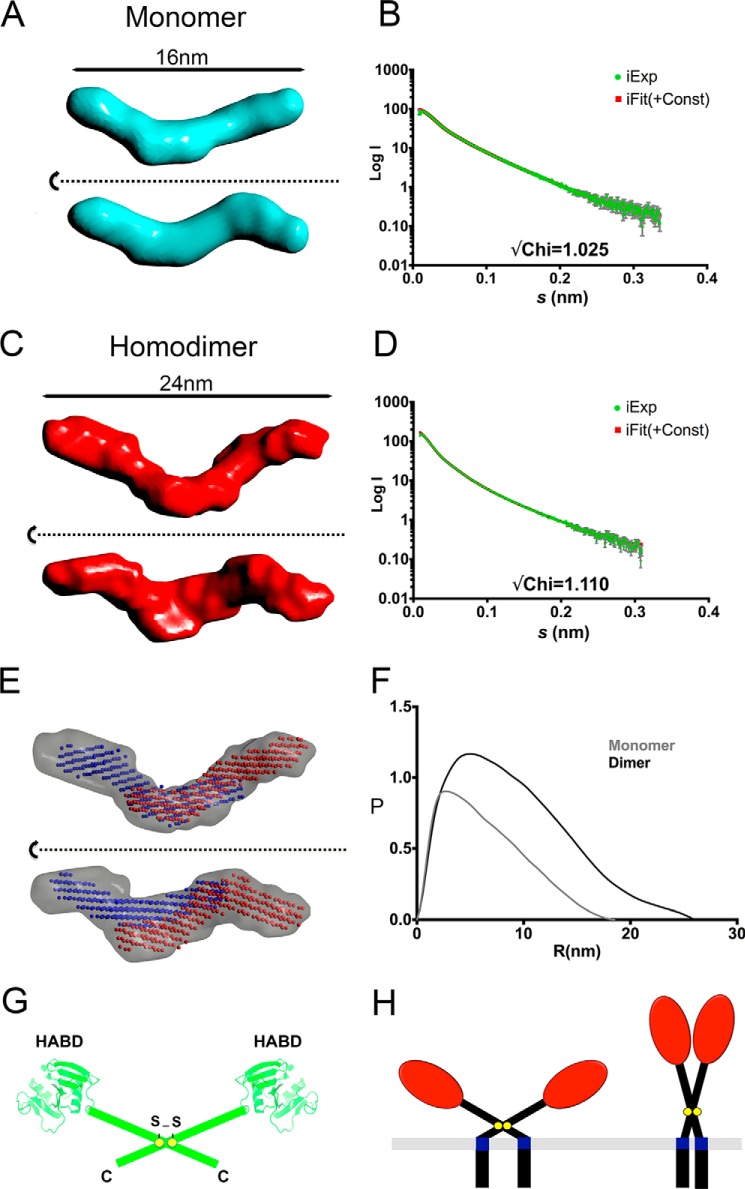
**SAXS analysis of LYVE-1 homodimer reveals an open-scissors conformation.** Soluble hLYVE-1 monomers and homodimers were subjected to small angle X-ray scattering analysis as described under “Experimental Procedures.” *A*, orthogonal views of the LYVE-1 monomer DAMMIF model, with fit of back-calculated scattering curves from the averaged model to raw scattering data (*B*), orthogonal views of the LYVE-1 dimer model (with no symmetry enforced) (*C*), with fit of back-calculated scattering curves from the averaged model to raw scattering data (*D*), orthogonal views of dimer envelope (*gray surface*) with monomer models (*red* and *blue spheres*) fitted inside using “Simultaneous Docking” function within Sculptor; SAXS envelope images produced using CCP4 mg (46) (*E*), comparison of derived P(r) distributions for monomer (*gray*) and dimer (*black*), calculated by GNOM (*F*), and schematic showing predicted open-scissors arrangement of the two monomer units within the LYVE-1 homodimer (*G*). The structure-based Link domain models are from Banerji *et al.* (10), and the membrane-proximal domains are depicted as rods. *H*, simplified schematic representing potential open and closed scissor arrangements of the homodimer (see text).

The magnitude of the increase in HA binding affinity and the observation of a 10-fold faster second step on-rate suggested that LYVE-1 homodimerization might juxtapose the two constituent HABDs in such a way as to synergize their interaction with the bound glycosaminoglycan. To obtain initial information on the three-dimensional structure of the LYVE-1 homodimer, we subjected purified preparations of LYVE-1 monomer and dimer to SAXS analysis. An evaluation of the resulting scattering data led to derivation of the *ab initio* models shown in Fig. 3, *A–C*, which indicated rod-like structures of ∼16 nm in length for the monomer and ∼24 nm for the homodimer, respectively, with a kink at one or both ends. The rod-like models are consistent with an extended conformation in the highly glycosylated membrane stalk region. This observation was further supported by vector length distribution analyses, which yielded highly asymmetric distributions in each case, typical of elongated structures (Fig. 3*F*). Fitting monomers to the model for the homodimer (Fig. 3*D*) indicated that the most likely arrangement is an anti-parallel orientation in which the two HA binding domains are located at opposite ends of the dimer with a considerable amount of overlap between the mid and C-terminal portions of the stalk region (Fig. 3*E*). Such an arrangement infers a degree of rotational freedom around the interchain disulfide at Cys-201 and an open-scissor like structure for the receptor dimer (Fig. 3*G*). Curiously, were this arrangement to be recapitulated in the full-length LYVE-1 homodimer at the cell surface, it would adopt a splayed, flattened structure with the HA binding domains lying closer to the surface of the plasma membrane than would be the case if the extracellular regions were arranged in an extended (closed scissors) parallel conformation (Fig. 3*H*).

##### LYVE-1 Homodimerization Is Obligatory for Stable HA Binding in Lymphatic Endothelial Cells

**FIGURE 4.**
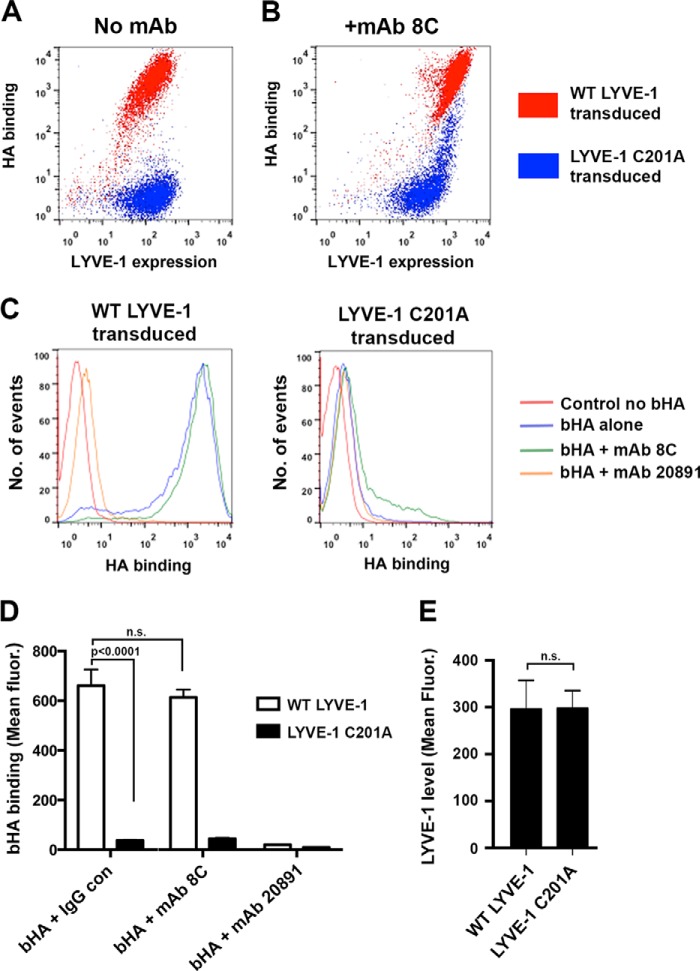
**Homodimerization of LYVE-1 is critical for avidity-dependent HA binding in lymphatic endothelial cells.** The particular requirement for LYVE-1 homodimers in supporting avidity dependent HA binding was assessed by flow cytometry of HDLEC lentivirally transduced with either wild-type or non-dimerizing C201A mutant LYVE-1, respectively, fused at the C terminus to emerald GFP. *A*, representative two-color FACS plot showing levels of HMW bHA binding as a function of LYVE-1 density in HDLEC overexpressing either wild-type LYVE-1 (*red*) or hLYVE-1 C201A non-dimerizing mutant (*blue*). *B*, corresponding two-color flow cytometry plot showing levels of HMW bHA binding as a function of LYVE-1 density in lentiviral transduced HDLEC after artificially induced cross-linking of LYVE-1 with bivalent mAb 8C. *C*, FACS histograms directly comparing the levels of HMW bHA binding in HDLEC transduced with either wild-type or C201A mutant LYVE-1 before and after treatment with LYVE-1 cross-linking mAb 8C or with HA blocking mAb 891 included as a control for specificity. *D*, quantitative comparison of bHA binding to HDLEC populations transfected with either wild-type hLYVE-1 or hLYVE-1 C201A non-dimerizing mutant before and after treatment with LYVE-1 mAbs (mean ± S.E. *n* = 3). *E*, confirmation of equivalent LYVE-1 surface expression in each case (mean ± S.E. *n* = 9). Statistical *p* values were obtained using the two-tailed unpaired *t* test (*n.s.*, non-significant). Data shown are from representative experiments that were repeated three times.

To determine whether the influence of LYVE-1 homodimerization on HA binding affinity was also manifest in the intact receptor in primary HDLEC, we transduced cells with either full-length wild-type LYVE-1 or the non-dimerizing C201A mutant in the lentiviral vector pHR using C-terminal GFP tagging to allow distinction of exogenous and endogenous receptor. Confirming our recent findings (13), overexpression of wild-type LYVE-1 by this method yielded sufficiently high receptor levels to support efficient binding of HMW bHA (Fig. 4*A*). Remarkably, however, equivalent expression of the C201A mutant yielded little if any bHA binding, demonstrating homodimer formation is critical for such function, even at high LYVE-1 densities (Fig. 4*A*). Furthermore, when the C201A supertransfectants were treated with the LYVE-1 cross-linking mAb 8C that triggers a dramatic increase in HA binding of wild-type (dimeric) receptor through receptor clustering (13), there was only a modest increase that was confined to the cell population with the very highest surface densities (Fig. 4, *B* and *C*). This population likely reflects cross-linking of the GFP-tagged C201A mutant and the less abundant endogenous wild-type homodimer.

These results provide firm evidence that the formation of LYVE-1 covalent homodimers is obligatory for stable HA binding in lymphatic endothelium. Importantly, they also imply that the Cys-201 intermolecular disulfide stabilizes a particular conformation within the homodimer that is critical for HA binding and that cannot be achieved through non-covalent monomer-monomer interactions, as non-dimerizing mutants were largely unable to engage the glycosaminoglycan, even when expressed at very high receptor densities, or artificially cross-linked with bivalent LYVE-1 mAbs.

##### The LYVE-1 Intermolecular Disulfide Is Highly Labile to Reduction

In light of the critical nature of the interchain disulfide for stable HA binding in HDLEC, we considered the possibility that its reversible formation and reduction might represent a mechanism for physiological regulation of HA binding, for example by changes in cellular redox potential. Such properties would predict that the Cys-201 residue is particularly labile, a property that has been reported for one of the conserved Link module disulfides in the related CD44 molecule (20). To address this possibility we used the reducing agent Tris (2-carboxyethyl) phosphine hydrochloride (TCEP-HCl) to probe the reactivity of the Cys-201 hydrophilic disulfide relative to the three conserved intrachain disulfides (Cys-36–Cys-139, Cys-61–Cys-128, and Cys-85–Cys-106) that stabilize the extended LYVE-1 HA binding domain.

**FIGURE 5.**
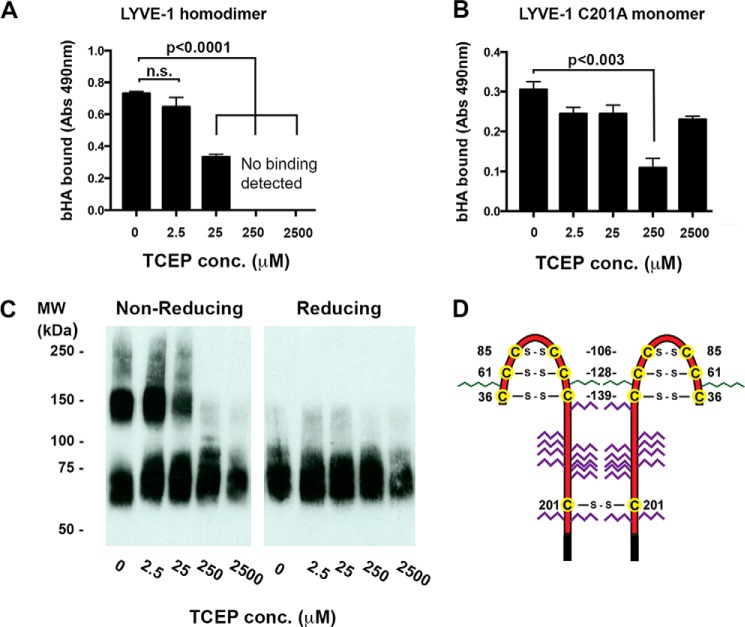
**The Cys-201 disulfide linkage in the LYVE-1 homodimer is highly labile.** The sensitivity to reduction of the Cys-201 intermolecular disulfide was investigated after exposure of soluble hLYVE-1 homodimers to varying concentrations of TCEP and assessment of the consequences for both HA binding and dimer disassembly (*A–C*) *A* and *B*, concentration-dependent effects of TCEP treatment on HMW bHA binding to immobilized hLYVE-1 (Δ238 His_10_) homodimers and hLYVE-1 (Δ238 His_10_) monomer (non-dimerizing C201A mutant), respectively, as detected with a streptavidin HRP conjugate. Values in each case are the mean ± S.E. *n* = 3. Statistics *p* values were obtained using the two-tailed unpaired *t* test (*n.s.*, non-significant). *C*, non-reducing SDS-PAGE analysis showing concentration-dependent effects of TCEP treatment on soluble homodimer disassembly assessed by Western blotting with LYVE-1 polyclonal antibody and green IRdye® 800 conjugate. *D*, schematic showing the location and numbering of individual cysteines (*yellow circles*) forming Link domain disulfides in the LYVE-1 homodimer; *N*- and *O*-linked glycan chains are colored *green* and *red*, respectively. Data shown are from representative experiments that were repeated three times.

In the first instance we assessed the effects on bHA binding of subjecting soluble wild-type LYVE-1 homodimer (LYVE-1 Δ238 His_10_) derived from transfected CHO cells to decades of increasing TCEP-HCl concentration in the range 2.5 μm–2.5 mm followed by alkylation of unreacted free thiols with *N*-ethylmaleimide. As shown in Fig. 5*A*, binding was largely unaffected at low concentrations of TCEP (2.5 μm) but was decreased ∼2-fold at 25 μm TCEP and completely abolished at concentrations of 250 μm and above. To ascertain whether this loss of binding was due to preferential reduction of the Cys-201 disulfide or involved co-incidental disruption of the structural disulfides within the LYVE-1 HA binding domain, we performed parallel TCEP reduction of the corresponding CHO-derived C201A mutant (LYVE-1 Δ238 His_10_), which, unlike its equivalent in HDLEC, retains some HA binding capacity (albeit ∼2-fold weaker). As shown in Fig. 5*B*, treatment of this mutant had little effect on bHA binding except for a consistent drop at 250 μm TCEP that was not apparent at higher concentrations (2.5 mm) and may possibly reflect disulfide rearrangement. To correlate the effects on HA binding with the degree of dimer disruption, we compared samples of LYVE-1 Δ238 His_10_ before and after TCEP-HCl reduction using non-reducing SDS-PAGE and Western blotting. As shown in Fig. 5*C*, disruption of homodimer again occurred at concentrations between 25 and 250 μm TCEP, the same range that led to ablation of bHA binding (Fig. 5*B*).

**FIGURE 6.**
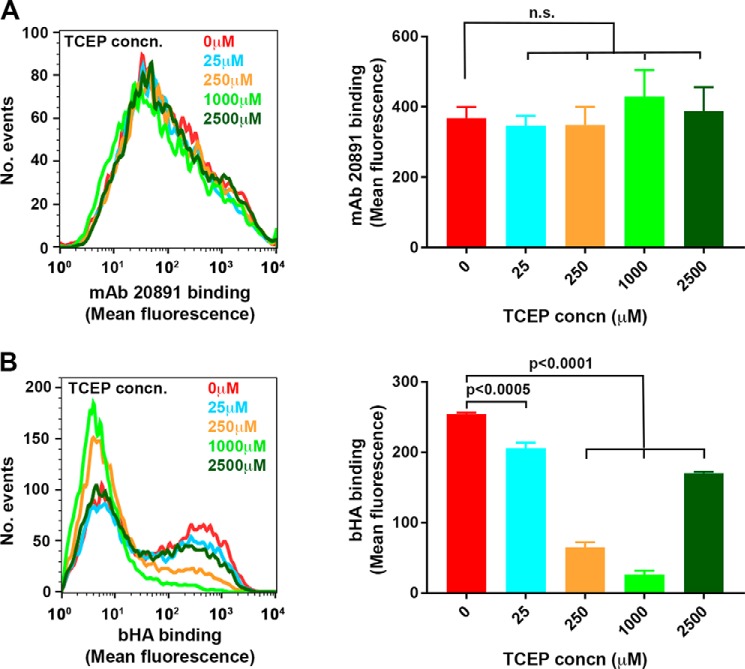
**Selective reduction of the LYVE-1 Cys-201 intermolecular disulfide ablates HA binding in transfected HDLEC.** The effects of treatment with the reducing agent TPEC on HA binding and receptor integrity in HDLECs lentivirally transduced with full-length hLYVE-1 was assessed by flow cytometry. *A*, FACS histogram and the associated *bar chart* showing the level of bHA binding to HDLEC after incubation with TCEP at the concentrations indicated. Binding was quantitated (mean ± S.E. *n* = 3) with streptavidin Alexa 647 conjugate. *n.s.*, non-significant. *B*, FACS histogram and the associated *bar chart* showing integrity of LYVE-1 HA binding domain at varying TCEP concentrations assessed by the levels of reactivity with LYVE-1 mAb 20891 (mean ± S.E. *n* = 3). Data shown are from a representative experiment that was repeated three times.

Next, to assess whether this selective lability of the Cys-201 intermolecular disulfide was manifest in the intact LYVE-1 molecule on the cell surface, we subjected samples of supertransfected HDLEC to selective reduction with increasing concentrations of TCEP-HCl followed by alkylation of free thiols with the cell-impermeable reagent methyl PEG12 maleimide. The results (Fig. 6*A*) show that just as with the soluble receptor, HA binding was almost fully abolished at a TCEP-HCl concentration of 250 μm with a partial recovery at levels of 2.5 mm. Furthermore, such reduction also blocked the ability of LYVE-1 cross-linking mAbs to potentiate bHA binding, similar to the results we obtained when the interchain disulfide was disrupted by site-directed mutagenesis (Fig. 4*B* and data not shown). Importantly, the TCEP concentrations that ablated HA binding had only marginal effects on the integrity of the HA binding Link module as assessed by reactivity with the LYVE-1 HA blocking mAb 20891 (Fig. 6*B*), arguing against a major contribution from overt off-target effects. Overall, these data indicate the Cys-201 disulfide that mediates LYVE-1 homodimer formation is highly labile and that its integrity is clearly critical for stable HA binding by LYVE-1 in lymphatic endothelium.

## Discussion

Here we have presented new evidence of a critical role for LYVE-1 disulfide-linked homodimerization in supporting avidity-dependent interactions with HA in lymphatic endothelium. Although the original cloning and sequencing of LYVE-1 cDNA had indicated the presence of an unpaired cysteine in the extracellular domain with a capacity to form homodimers, neither the phenomenon nor its physiological significance has been explored. Our present manuscript has revealed that native LYVE-1 is expressed predominantly as homodimers in primary lymphatic endothelial cells cultured *in vitro* as well as in lymphatic vessels present within skin tissue *in vivo* and that such dimers are highly labile to disassembly by changes in redox conditions. Moreover, they have established that cysteine 201 in the stalk region of LYVE-1 is the residue critical for disulfide-linked dimer formation, excluding possible involvement of the free cysteine residue C in the transmembrane domain whose counterpart in the closely related HA receptor CD44 has been reported to mediate limited self-association in response to phorbol ester stimulation (21, 22). Importantly, our studies also reveal that homodimerization leads to a substantial (15× fold) increase in the experimentally determined HA binding affinity of the receptor, underlining the potential for the process to act as a mechanism for tuning physiologically important interactions between LYVE-1 and its glycosaminoglycan ligand *in vivo*.

The absence of any significant HA binding in LECs is curious in view of our finding that they express predominantly LYVE-1 homodimers that have appreciable affinity for the ligand (*K_D_* 8.2 μm). Clearly, as we recently showed that firm interactions between LYVE-1 and HA are strictly regulated by avidity (13). Thus, to achieve such binding in native LECs, the HA polymer must first be organized within cross-linked protein complexes such as TSG-6·HA or as a dense glycocalyx such as the group A streptococcal HA capsule (13, 14). Likewise to attain stable binding of free uncomplexed HA, the levels of LYVE-1 must be raised above a critical threshold density as demonstrated by engineered overexpression or antibody-driven receptor clustering (13). One interpretation of these findings is that constraints on LYVE-1 lateral mobility limit free HA polymers from harnessing the required avidity in native LECs by hindering engagement with a sufficient number of homodimers for firm binding. Indeed preliminary support for this notion comes from our observations that LYVE-1 preferentially associates with the detergent-insoluble cytoskeleton fraction in LECs^3^ and that HMW HA binding is enhanced when native LEC are treated with actin depolymerizing agents.^4^

Nevertheless, it is evident that factors additional to avidity must also contribute to the distinctive binding properties of LYVE-1 homodimers. It was particularly notable that disruption of the intermolecular disulfide in the C201A-mutated receptor completely ablated its capacity to support HMW HA binding in lentiviral transduced HDLEC even when expressed at surface densities far above the critical threshold required for binding by normal dimerization-competent LYVE-1. Furthermore, in marked contrast to the native receptor in LECs, there was little or no enhancement of HMW HA binding when the dimer-deficient mutant transfectants were subjected to cross-linking with bivalent LYVE-1 antibodies. We interpret these results to indicate that the native LYVE-1 homodimer is linked through the Cys-201 disulfide bond in a manner that cannot be mimicked by antibodies that bridge the N-terminal HA binding domains. Rather than merely linking the two partners in the LYVE-1 homodimer, the intermolecular disulfide appears to have a more specific impact on the conformation of the receptor. This notion is also supported by our structural studies with purified soluble LYVE-1 using SAXS, which revealed that the homodimer adopts an open scissor-like configuration with the disulfide at the hinge and an HABD on each tip. It is further supported by our Biacore analyses, which showed that the second step on-rate for HA binding to the homodimer is ∼100× faster than the first step on-rate. The mutual disposition of the two subunit HABDs that is adopted by the native homodimer may for example allow recognition of a low energy long range HA conformation that is absent in free uncomplexed configurations of the glycosaminoglycan. Indeed the anti-parallel arrangement of the LYVE-1 HABDs would render them unable to interact with contiguous regions of the same HA strand without the introduction of an intervening loop, which would be entropically unfavorable. Interestingly, however, it would favor interactions with anti-parallel HA chains in the context of cross-linked complexes and provide a potential explanation for how LYVE-1 can have selective affinity for cross-linked HA.

Conceivably, the splayed arrangement of the full-length membrane-anchored LYVE-1 homodimer indicated by our SAXS analyses would also impose a complementary separation between the two cytoplasmic tails. Indeed, our failure to detect significant interaction between adjacent LYVE-1 C termini in transfected 293T cells using bioluminescent resonance energy transfer (BRET) in earlier studies would be consistent with this arrangement (10). One particularly attractive possibility is that the LYVE-1 homodimer undergoes conformational change upon binding HA that alters the spacing of the HABDs to optimize their mutual interaction with ligand.

Intriguingly, our studies using the efficient reducing agent TCEP-HCl showed that the Cys-201 intermolecular disulfide in the LYVE-1 homodimer is highly labile. Notably, this particular disulfide underwent preferential reduction at TCEP concentrations (≤250 μm) well below those required to break the structural disulfides that are critical for the stability of the extended Link module as evidenced from comparisons with the non-dimerizing LYVE-1 C201A mutant. Accordingly, these relatively low TCEP concentrations caused complete dimer disassembly and substantial loss of HA binding without overtly disrupting Link module conformation, as assessed by monoclonal antibody reactivity. The selective lability of the Cys-201 intermolecular disulfide is also supported by initial quantitative mass spectrometric analyses of the TCEP modified receptor,^5^ which suggests an almost 10-fold higher reactivity of Cys-201 in relation to the three structural disulfides (Cys-36–Cys-39, Cys-61–Cys-128, and Cys-85–Cys-105) that stabilize the extended Link module. This marks an interesting contrast with CD44 in which the Link disulfide Cys-77–Cys-97 was recently shown to act as a redox-regulated switch for HA binding using similar methodologies (20). Such findings are consistent with the notion that Cys-201 occupies an exposed environment on the surface of LYVE-1 where changes in extracellular redox conditions could potentially alter homodimer formation and have a dramatic influence on HA binding.

The presence of labile disulfides has been described for a number of biologically important cell surface receptors, and the phenomenon of redox regulation of protein function is now becoming firmly established (23, 24). For example, reduction of a labile disulfide in CD132, the common γ chain of the IL-2 receptor, inhibits IL-2 signaling in cells (25), whereas reduction of key disulfides in the platelet αIIb/βII integrin has been reported to enhance binding of the ligand fibrin leading to increased thrombus formation (26, 27). The stimulus for such disulfide modulation can arise during inflammation or cell activation through the cellular secretion of glutathione in addition to protein disulfide isomerases and the disulfide oxidoreductase thioredoxin (for review, see Ref. 25). Indeed the secretion of these anti-oxidants by activated dendritic cells is thought to exert redox regulation of T cell activation and proliferation within the reducing extracellular environment necessary for immune responses (28, 29). Our studies here showed that LYVE-1 homodimers, which constitute the predominant species on the surface of human dermal LEC, were highly susceptible to reduction during *in vitro* cell lysis, in a process that could be blocked by the thiol modifying agent NEM. This indicates that LECs may contain high levels of endogenous thiol-based anti-oxidants, which could in principle influence the stability of homodimers *in vivo*. Moreover, we found that LYVE-1 in mouse skin and lymph nodes occurs almost exclusively as dimers, whereas almost 30% of the receptor occurs as monomers in liver and lung. This provides evidence that the relative levels of each form of the receptor may be regulated by tissue resident redox conditions. However, as LYVE-1 expressed mainly in lymphatics in the former tissues and blood vessels in the latter (4, 11), it is also likely that homodimerization is partly influenced by innate differences in receptor biosynthesis among lymphatic and vascular endothelia. It is also worth noting that the Cys-201 intermolecular disulfide lies upstream of the proteolytic cleavage site (Phe-226–Glu-229) by which soluble LYVE-1 ectodomains are shed by ADAM-17 in response to VEGF-A or phorbol ester (30). An important function may, therefore, be to preserve receptor bivalency and high affinity HA binding, once LYVE-1 is released from the plasma membrane. Clearly, additional studies will be required to evaluate these possibilities and also determine whether the stability of LYVE-1 homodimers and HA binding affinity is subject to further dynamic regulation in response to inflammation or other physiologically induced changes in the extracellular redox environment.

**FIGURE 7.**
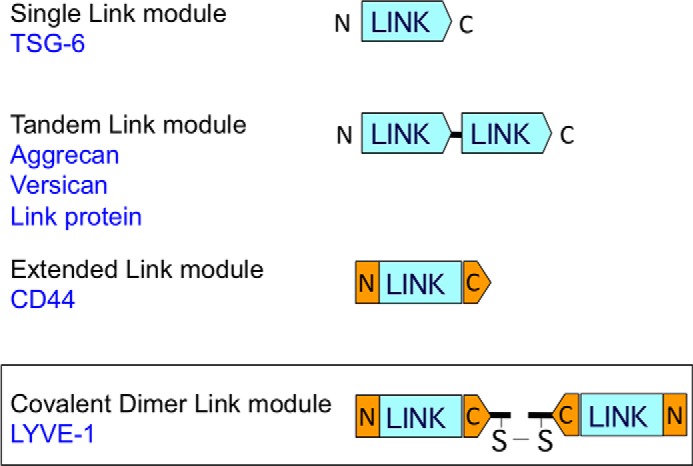
**LYVE-1 marks a novel category of Link superfamily proteins with covalently joined Link modules.** The different arrangements of Link modules within the three currently recognized categories of Link protein superfamily HA-binding proteins are shown together with a new fourth category containing covalently paired Link modules, of which LYVE-1 is the only known member to date. Examples from each of the other categories are listed in *blue*. Note that the N- and C-terminal extensions to the consensus Link module in CD44 have predicted counterparts in LYVE-1, and that unlike the tandem Link module category, the LYVE-1 covalent dimer Link modules are predicted to have a mutually antiparallel orientation.

Finally, the covalent association of HA binding Link modules on separate polypeptide chains in the manner reported here for LYVE-1 is so far unique among members of the Link module superfamily (Fig. 7). The large HA binding proteoglycans aggrecan and versican each contain dual HABDs within their N-terminal G1 structural domains (31). Importantly, in both cases these occur in tandem in an arrangement that increases their binding affinity for the same individual HA chain rather than separate antiparallel HA chains as discussed above for LYVE-1 homodimers. We also note that an analogous disulfide-linked homodimerization of carbohydrate binding domains is a feature of the small subset of the wider C-type lectin-like domain (CTLD) superfamily to which the Link module is structurally related (7, 12, 32) and which includes CD69, Lox-1, the A33 poxvirus receptor, and the Ly49 and NKG2/CD94 families of NK cell receptors (33). However, in contrast to LYVE-1, the intermolecular disulfides in these latter receptors are, to varying degrees, dispensable for dimer formation as the partner proteins can undergo non-covalent self-association. Thus far we have not observed any tendency for recombinant soluble LYVE-1 HABDs to dimerize noncovalently, and C201A full ectodomain constructs migrate solely as monomers in size exclusion chromatography (data not shown). Moreover, unlike receptors such as Lox-1 (34), the interchain disulfide of LYVE-1 has been highly conserved throughout evolution, and Cys-201 is present even in avian and reptile LYVE-1 sequences (not shown). The capacity to form the intermolecular disulfide would, therefore, appear to be an evolutionary adaptation of significant importance to LYVE-1 function. It is interesting to note that acquisition of the critical free cysteine in LYVE-1 by reptiles/birds and mammals appears after their evolutionary branching from fish and amphibians (which both lack the residue), and hence dimer formation segregates with the emergence of more developed lymphoid organs and cell trafficking. A requirement for dimerization may also be rationalized on the basis that lymphatics within the tissues encounter many different configurations of HA in fulfilling their function as conduits for glycosaminoglycan transport and breakdown in draining lymph nodes. Indeed lymph fluid contains relatively high concentrations of HA (up to 20 μg/ml), which are at least 2 orders of magnitude greater than those of blood (35). This is likely to comprise many different configurations including “free” HA generated by local matrix turnover, large HA-protein complexes deposited in tissues during inflammation, and HA within the surface glycocalyx of migrating leukocytes or even invading bacterial pathogens (5, 14, 36–38). As we have shown recently, only HA that is organized in complexes can bind LYVE-1 constitutively in native HDLEC (13), and the predominance of higher affinity homodimers in such cells may well allow discrimination between these preferred HA configurations and bulk ligand through a mixture of avidity and conformation recognition. Moreover, the potential to disassemble the higher affinity homodimers in response to changes in redox potential and anti-oxidant production would allow lymphatic endothelial cells to engage and disengage with either HA complexes or leukocyte glycocalyx in an appropriate manner during inflammation and its resolution. These and other aspects of LYVE-1 biology are currently under investigation in our laboratory.

## Experimental Procedures

### Antibodies

The following LYVE-1-specific antibodies were used: polyclonal goat anti-human (AF20891, R&D Systems), a biotinylated polyclonal (BAF20891, R&D Systems), and the mAbs 20891, 20982, 20893 (all R&D Systems), 6A, 8C, and 3A (all made in-house). A rabbit polyclonal specific for human ERK1 and -2 was purchased from Sigma.

### Biotinylated HA

bHA was prepared from HMW rooster comb HA (Sigma) using a procedure modified from that detailed in Yang *et al.* (39). Briefly, HA was dissolved in 0.1 m MES, pH 5.5, at 5 mg/ml and combined with *N-*(3-dimethylaminopropyl)-*N*′-ethylcarbodiimide hydrochloride (EDAC) dissolved in 0.1 m MES, pH 5.5, at a final concentration of 162.5 μg/ml before biotin-LC-hydrazide (Pierce) in DMSO was added to a final concentration of 1 mm and stirred overnight at room temperature. The resulting labeled HA was dialyzed extensively against H_2_O containing 0.05% (w/v) NaN_3_.

### Cell Culture

HDLECs were prepared by LYVE-1 MACs® bead immunoselection from surgically excised tissue samples using individual anonymized donors undergoing elective procedures for breast reduction and abdominoplasty at the John Radcliffe Hospital, Oxford as described previously, with full UK ethical approval (40). The cells were obtained from a total of nine such donors, frozen in aliquots at early passage, and subsequently thawed and maintained in culture for up to six passages. Briefly, skin was digested overnight at 4 °C with 2 mg/ml Dispase (Invitrogen) in PBS, pH 7.5, to remove the epidermis. Dermal cells were released from tissue by scraping, passed through a 70-μm cell strainer (BD Biosciences), and cultured in EGM-2 MV medium in flasks that had previously been coated in 0.1% gelatin (Sigma) in PBS. Culture was carried out at 37 °C, 5% CO_2_ in a humidified atmosphere. Adherent dermal cells were lifted with Accutase (PAA Laboratories) and immunoselected with the mouse anti-human LYVE-1 mAb 8C followed by magnetic retrieval with anti-mouse MACS® beads (Miltenyi Biotec). The resulting cells were cultured in EGM-2 MV using plastic tissue culture flasks that had been precoated with 0.1% gelatin (Invitrogen). Individual experiments were performed with confluent LECs from one donor rather than mixtures, but each repeat involved the use of LECs isolated from a different donor to accommodate minor variations in LYVE-1 expression and, thus, represented true biological replicates.

### LYVE-1 Constructs for Cell Surface Expression

The coding sequence was amplified from full-length cDNA using the high fidelity polymerase pfu Ultra AD (Agilent) with the following primers: hLYVE-1–14 MluI forward (5′-GCGACGCGTGAAGGGGTAGGCACGATGGCCAGG; hLYVE-1 969* NotI reverse (5′-CGCGCGGCCGC*CTA*AACTTCAGCTTCCAGGCATCGCAC). Segments of the primers that generate restriction sites are underlined, and the stop codon is denoted by italics in the reverse primer.

Similarly, the coding sequence of LYVE-1 was fused at its C terminus with enhanced green fluorescence GFP. The fragment was amplified using hLYVE-1–14 MluI F and the reverse primer: hLYVE-1 969 BamHI reverse (5′-CGGGATCCAACTTCAGCTTCCAGGCATCGCAC).

The amplified product was cloned into the lentiviral vector pHR Sin, based on the HIV retrovirus. 293T cells were transiently transfected with pHR Sin plasmids together with the packaging plasmids pMD.G and p8.91 (41) in six-well plates using Genejuice (Merck) according to the manufacturer's instructions. Growth medium was changed immediately before transfection to either EGM-2 MV medium (Lonza) for compatibility with primary HDLECS or DMEM supplemented with 10%FCS for 293T cells. Packaged lentivirus was then harvested from culture supernatants at 48–72 h post-transfection, passed through a 0.45-μm filter to remove cell debris and subsequently used to transduce 293T or primary HDLEC (2 × 10^5^ cells). Transduced cultures were incubated overnight before the supernatants were replaced with fresh growth medium. In some cases cells positive for LYVE-1 or expressing a desired level of LYVE-1 were selected by fluorescence activated cell sorting (FACS).

### LYVE-1 Constructs for Expression as Soluble Ectodomains

The full-length extracellular domain (terminating after residue number 238) from wild-type LYVE-1 was amplified from a plasmid carrying human LYVE-1 cDNA using the high fidelity polymerase pfu Ultra AD (Agilent) with the following primers: hLY-14HindIII forward (5′-GCGAAGCTTGAAGGGGTAGGCACGATGGCCAGGTG-3′; hLY+71410His*XbaI reverse 5′CGCTCTAGA***TTA***ATGGTGATGGTGATGGTGATGGTGATGGTGCGTGGGGACACCTCCAAACCC, where the reverse primer encodes a 10 histidine (His_10_) tag and stop codon, italicized in bold.

The PCR product was digested with the restriction endonucleases HindIII and XbaI and ligated into the expression vector pEE14. An error-free clone was selected after sequencing and transfected into CHO cells using the FuGENE lipid transfection reagent (Roche Applied Science). Clones recombinant for the pEE14 plasmid were selected in media containing 40 μm methionine sulfoxamine, and a clone was selected after a Western blot of supernatants probed with a LYVE-1-specific polyclonal antibody. This clone was stored and later used for the production of wild-type hLYVE-1 Δ238 His_10_ recombinant protein.

An equivalent amplified product was made from a mutated version of the LYVE-1 cDNA carrying the mutation C201A using the following primers: hLY-14BamHI forward (5′-GCGGGATCCGAAGGGGTAGGCACGATGGCCAGGTG);hLY71410His*XhoI reverse (5′GCGCCTCGAG***TTA***ATGGTGATGGTGATGGTGATGGTGATGGTGCGTGGGGACACCTCCAAACCC, where the reverse primer encodes a His_10_ tag and stop codon italicized in bold.

The amplified fragment was cloned into a variant of the pHR SIN vector carrying an internal ribosome entry site (IRES) upstream of a gene encoding emerald GFP. Production of virus-like particles and transduction of CHO cells were as described above. After 5 days, cells with high GFP expression were selected by FACS and stored as a stably transfected cell line for the production of human C201A hLYVE-1 Δ238 His_10_ recombinant protein.

### Recombinant Protein Purification

The transfected clone for hLYVE-1 Δ238 His_10_ was expanded at 37 °C, 5% CO_2_ in high glucose Dulbecco's modified Eagle's medium (DMEM; Life Technologies) supplemented with 10% fetal calf serum (FCS) dialyzed against PBS (First Link Ltd., Wolverhampton, UK), 1 mm sodium pyruvate (Life Technologies), 1× GS medium supplement (EMD Millipore, supplied at 50×), 1× penicillin/streptomycin (Sigma, supplied at 100×), and 40 μm l-methionine sulfoximine (Sigma). On reaching confluence in 15-cm tissue culture Petri dishes, the medium was replaced by serum-free DMEM supplemented with 1 mm sodium pyruvate, 2 mm sodium butyrate, and 1× penicillin/streptomycin. After a further 4 days, tissue culture supernatant was aspirated and passed over a 0.22-μm filter before diluting 1:3 in PBS. Imidazole, pH 7.4, and NaCl were added to give final concentrations of 20 and 300 mm, respectively. His-tagged protein was extracted by passing the diluted and supplemented supernatant over a 5-ml His Trap column (GE Healthcare) before washing (PBS, 20 mm imidazole, supplemented with a further 150 mm NaCl) and then eluting (PBS, 500 mm imidazole, supplemented with a further 150 mm NaCl). After elution, the protein was buffer-exchanged into PBS and concentrated before size exclusion chromatography on a Superdex 200 10/300GL column (GE Healthcare). The transfected line for hLYVE-1 C201A Δ238 His_10_ was treated identically, except that cells were expanded in DMEM supplemented with 10% FCS, 1 mm sodium pyruvate, 1× penicillin/streptomycin, and 1× l-glutamine (Sigma, supplied at 100×). Yields for both the wild-type LYVE-1 and C201A recombinant proteins were typically 4–8 mg/liter of tissue culture supernatant.

### Western Blot Analysis of Cell and Tissue Lysates and Soluble Recombinant LYVE-1 Proteins

Samples of primary HDLEC, LYVE-1-transfected cell lines, or purified LYVE-1 proteins were lysed or dissolved (98 °C, 5 min) in LDS sample buffer (Invitrogen) containing 5 mm NEM with or without 2-mercaptoethanol (5%, v/v) for reducing or non-reducing SDS-PAGE as appropriate. In the case of mouse tissues, these were first minced in PBS, pH 7.5, containing Complete^TM^EDTA-free protease inhibitor mixture and 5 mm NEM before lysis in LDS sample buffer using a Dounce homogenizer, then centrifuged (11,000 × *g*, 5 min) and sonicated (4 °C) to shear genomic DNA. All samples were then electrophoresed on Novex® 4–12% Tris-Glycine Mini Protein gels followed by transfer to Immobilon-Fl PVDF membranes. Blots were then probed with the human or mouse LYVE-1-specific polyclonal antibodies AF2089 or AF2125, respectively, and detected with an anti-goat IRdye® 800 conjugate for quantitative imaging using a Licor Odyssey scanner and Image Studio software. In some cases 10-μg samples of unfractionated WT LYVE-1 His_10_ were treated with TCEP-HCl at concentrations ranging from 25 to 2500 μm before alkylation with NEM. Such treated samples were analyzed by Western blot as described above.

### Selective Reduction of Labile LYVE-1 Disulfide Bonds in Cultured Primary HDLEC

LYVE-1 transfected HDLECs were lifted and washed in 10% FCS and 0.05% sodium azide in PBS. After counting, aliquots of 2 × 10^5^ cells were subjected to reduction with varying concentrations (2.5 μm–2.5 mm) of TCEP-HCl for 30 min at room temperature. Resulting sulfhydryl groups were then alkylated with 5 mm methyl PEG12 maleimide (Pierce) for 30 min at room temperature.

### Selective Reduction of Labile Disulfide Bonds in Soluble LYVE-1 Homodimers

Dimeric wild-type hLYVE-1 Δ238 His_10_ was purified by size exclusion chromatography using a Superdex 200 10/300GL column. Aliquots (10 μg) were treated with 0–2.5 mm TCEP-HCl for 30 min at room temperature before “kinetically trapping” cysteines liberated from reduced disulfide bonds through alkylation with 5 μm-5 mm NEM as appropriate.

### Surface Plasmon Resonance

A series S streptavidin sensor chip (GE Healthcare) was coated with 300 kDa (“Select”) hyaluronan biotinylated at the reducing end (Hyalose). Analytes were buffer-exchanged into HEPES-buffered saline (10 mm HEPES, pH 7.4, and 150 mm NaCl), which was supplemented with EDTA (2 mm) and P20 detergent (0.02% v/v). Dimer was prepared by multiple rounds of size exclusion chromatography of soluble wild-type hLYVE-1, whereas monomer was prepared by size exclusion chromatography of the soluble ectodomain carrying the C201A mutation to prevent any conversion to dimer during the binding analysis. Initial sensorgrams indicated that binding constants for the monomer could best be derived from equilibrium binding experiments. In contrast, because of the extended time required for homodimer binding to reach equilibrium, single cycle kinetic analysis was used to derive kinetic constants. Concentrations of dimeric LYVE-1 of 12.8, 64, and 320 nm were sequentially injected where each injection was 180 s followed by a 60-s dissociation period except for the final dissociation, which was 600 s. LYVE-1 monomer was injected at concentrations of 128, 64, 32, 16, 8, 4, 2, 1, and 0.5 μm with a regeneration step of an injection of 0.1 m glycine, pH 2.5, between each of the LYVE-1 injections. All data were collected on a Biacore T200 system at 25 °C and analyzed using the BIAevaluation software package. Methods for modeling and analysis of monovalent and bivalent hLYVE-1 binding to hyaluronan are detailed in the supplemental information.

### Microtiter Plate HA Binding Assays

#### 

##### Standard Assay

Nunc Maxisorb microtiter plates were coated with purified hLYVE-1 monomer or dimer species, prepared as described above, each at a concentration of 4 μg/ml in PBS at room temperature overnight. Plates were then blocked (with 1% w/v BSA in PBS, 0.05% Tween 20) before the addition of bHA in 2-fold dilutions from 5 μg/ml to 39.06 ng/ml. Streptavidin HRP (Pierce) was added to detect bound bHA before developing the plate with *o*-phenylenediamine substrate (Sigma) and quenching with 1 m H_2_SO_4_. Absorbance at 490 nm was recorded using an i-Mark^TM^ plate reader (Bio-Rad).

##### TCEP-HCl Treatment

Nunc Maxisorb microtiter plates were coated with purified monomeric C201A LYVE-1 8Δ His_10_ or dimer purified from expression of WT LYVE-1 Δ238 His_10_ at a concentration of 40 μg/ml to ensure detectable HA binding for both species. Bound LYVE-1 was treated with 0, 2.5, 25, 250, or 2500 μm TCEP for 15 min at room temperature followed by alkylation of free thiols using 5 mm NEM. Additionally, control wells coated with LYVE-1 received neither TCEP nor NEM treatment. HA binding was determined for both monomer and dimer by the addition of biotinylated HMW HA at 5 μg/ml. Streptavidin HRP was added to detect bound bHA, and after color development, plates were quenched with H_2_SO_4_ as before. Absorbance at 490 nm was recorded using an i-Mark^TM^ plate reader (Bio-Rad).

### SAXS Analysis and Molecular Modeling

Samples of monomeric and dimeric LYVE-1 were produced as outlined above. SAXS data were collected at beamline X-33, DESY, Germany, at concentrations of between 9.2 and 4.6 mg/ml (monomer) and 6.6 and 4.6 mg/ml (dimer). Scattering data from matched buffer solutions were subtracted using the program PRIMUS (42), and the radius of gyration, forward scattering intensity, and distance distribution function p*(r*) were evaluated with the program GNOM (43). Particle shapes were generated from the experimental scattering profiles using *ab initio* modeling with DAMMIF (44). Multiple DAMMIF simulations (>20) were performed for both monomer and dimer (with and without *p*2 symmetry constraints for the dimer), and these generated very similar but not identical shapes in each case. Enforcing *p*2 symmetry did not appreciably alter the shape or improve the fit to the raw data, so all dimer models presented are those created without *p*2 symmetry. An averaged filtered shape was generated using DAMAVER and DAMFILT (45).

## Author Contributions

D. G. J. conceived and coordinated the study, interpreted the data, and co-wrote the manuscript. S. B. conceived the major elements of the study, designed, performed, and critically analyzed the experiments, and co-wrote the manuscript. W. L. designed, performed, and critically analyzed the experiments using LYVE-1C201A mutants. D. C. B. and A. J. D. collected, processed, analyzed, and interpreted the SAXS data and derived the molecular models. C. M. conceived, designed, analyzed, and interpreted the kinetic trapping and disulfide lability assays. A. K. performed the Western blotting analyses. O. D. and P. A. v. d. M. processed and re-analyzed the Biacore binding data and derived the binding constants. All authors reviewed the data and approved the final version of the manuscript.

## Supplementary Material

Supplemental Data

## Footnotes

3 D. Markovic and D. G. Jackson, unpublished information.

4 T. Stanly, D. G. Jackson, and C. Eggeling, unpublished information.

5 S. Banerji, C. Metcalf, and D. G. Jackson, unpublished information.

## Footnotes

2 The abbreviations used are:

HA: hyaluronan
HMW: high molecular weight
NEM: *N*-ethylmaleimide
SAXS: small angle X-ray scattering
HDLEC: human dermal endothelial cells
bHA: biotinylated HA
TCEP: Tris (2-carboxyethyl) phosphine
hLYVE-1: human lymphatic vessel hyaluronan receptor-1
LEC: lymphatic endothelial cell
HABD: hyaluronan binding domain.
