# Supplementary material for: Homodimerization of the Lymph Vessel Endothelial Receptor LYVE-1 through a Redox-labile Disulfide Is Critical for Hyaluronan Binding in Lymphatic Endothelium
Source: J Biol Chem. 2016 Oct 12;291(48):25004–18. doi: 10.1074/jbc.M116.736926 (PMC5122770; doi:10.1074/jbc.M116.736926)
Supplement: Supplemental Data [file supp_291_48_25004__index.html]

Homodimerisation of the Lymph Vessel Endothelial Receptor LYVE-1 through a Redox-Labile Disulfide is critical for Hyaluronan Binding in Lymphatic Endothelium — Homodimerization of the Lymph Vessel Endothelial Receptor LYVE-1 through a Redox-labile Disulfide Is Critical for Hyaluronan Binding in Lymphatic Endothelium — Function of LYVE-1 Disulfide-linked Homodimers — Supplemental Data 

# Homodimerization of the Lymph Vessel Endothelial Receptor LYVE-1 through a Redox-labile Disulfide Is Critical for Hyaluronan Binding in Lymphatic Endothelium

## Supplemental Data

- Supplemental section (.pdf, 140 KB) - Determination of binding constants
