# Supplementary material for: Homodimerization of the Lymph Vessel Endothelial Receptor LYVE-1 through a Redox-labile Disulfide Is Critical for Hyaluronan Binding in Lymphatic Endothelium
Source: J Biol Chem. 2016 Oct 12;291(48):25004–18. doi: 10.1074/jbc.M116.736926 (PMC5122770; doi:10.1074/jbc.M116.736926)
Supplement: Supplemental Data [file 10.1074_M116.736926_jbc.M116.736926-1.pdf]

Supplementary Information: Disulfide linked homodimers of the lymph vessel endothelial receptor LYVE-1 are critical for avidity dependent hyaluronan binding in lymphatic endothelium

Suneale Banerji[1], William Lawrance[1], Clive Metcalfe[2], David C. Briggs[3], Akira Yamauchi[4]
Omer Dushek[2], P. Anton van der Merwe[2], Anthony J. Day[3], and David G. Jackson[1]

[1]Medical Research Council Human Immunology Unit, Weatherall Institute of Molecular Medicine, John Radcliffe Hospital, Headington, Oxford OX3 9DS, U.K

[2]Sir William Dunn School of Pathology, University of Oxford, South Parks Road, Oxford, OX1 3RE, U.K.

[3]Wellcome Trust Centre for Cell Matrix Research, Faculty of Biology, Medicine and Health, University of Manchester, Oxford Road, Manchester, M13 9PT, U.K.

[4]Department of Biochemistry, Kawasaki Medical School, 577 Matsushima, Kurashiki 701-0192, Japan

Contents

1 Fitting bivalent binding kinetics **2**

2 Effective binding parameters **3**

2.1 Effective binding affinities 3

2.1.1 Monovalent interaction 3

2.2 Bivalent interaction 3

2.3 Effective binding kinetics 4

2.3.1 Monovalent interaction 4

2.3.2 Bivalent interaction 4

3 Estimating surface concentration of HA **5**

1 Fitting bivalent binding kinetics

Fitting monovalent binding data, such as monomeric LYVE-1 binding to surface immobilised HA (Fig. 3B), is a straightforward analysis commonly carried out on SPR binding data. Fitting bivalent binding data, such as homodimeric LYVE-1 binding to surface immobilised HA (Fig. 3C), is more complicated and care must be taken to avoid over-fitting.

The bivalent analyte binding model (provided in the BIAcore T200 evaluation software) contains four fitting parameters for the following reactions:

$$A + R \underset{k_{\text{off}}^1}{\overset{2k_{\text{on}}^1}{\rightleftharpoons}} R_1 \tag{1}$$

$$A + R_1 \underset{2k_{\text{off}}^2}{\overset{k_{\text{on}}^2}{\rightleftharpoons}} R_2 \tag{2}$$

where k_{on}^1 is the solution bimolecular on-rate (in units of $\mu\text{M}^{-1}\text{s}^{-1}$), k_{on}^2 is the surface bimolecular on-rate (in units of $\text{RU}^{-1}\text{s}^{-1}$), k_{off}^1 and k_{off}^2 are the off-rates (in units of s^{-1}) when the LYVE-1 homodimer is bound monovalently and bivalently. We found that fitting this model with 4 free parameters produced an excellent fit but that the fitted parameters were not unique so that a repeated fit (with different initial conditions) produced an equally good fit but with a completely different set of parameters. This is a hallmark of over-fitting.

To avoid the problem of over-fitting, we made several simplifications. First, we assumed that the two off-rates are identical (${k^1}_{\text{off}} = {k^2}_{\text{off}}$) and, given that the monovalent K_D has already been determined (Fig. 2B), we assumed that the solution bimolecular on-rate is $k_{\text{on}}^1 = k_{\text{off}}^1/(124\mu\text{M})$. These two assumptions reduce the number of fitting parameters from four (k_{on}^1, k_{on}^2, k_{off}^1, k_{off}^2) to two (k_{on}^2, k_{off}^1). Fitting this 2-parameter model to the experimental data produced $k_{\text{on}}^2 = 37.3\ \text{RU}^{-1}\text{s}^{-1}$ and $k_{\text{off}}^1 = 8.55\ \text{s}^{-1}$ with an excellent fit (Fig. 3C). Moreover, we obtained identical parameter values with repeated fits (with different initial conditions) suggesting that the parameter values are unique and that we are not over-fitting the experimental data.

We can convert the second step on-rate (k_{on}^2) into more familiar units by noting that in general 1 mg/ml protein gives 100 RU. It follows that $k_{\text{on}}^2 = 7.46\ \mu\text{M}^{-1}\text{s}^{-1}$.

In summary, this analysis has revealed that the LYVE-1 interaction with HA can be captured with a bivalent analyte model with a solution bimolecular on-rate of $k_{\text{on}}^1 = 0.0692\ \mu\text{M}^{-1}\text{s}^{-1}$, a surface bimolecular on-rate $k_{\text{on}}^2 = 7.46\ \mu\text{M}^{-1}\text{s}^{-1}$, and off-rates $k_{\text{off}}^1 = k_{\text{off}}^2 = 8.55\ \text{s}^{-1}$.

In the following sections we derive formulae for effective parameters that can be used to compare monovalent and bivalent LYVE-1 interaction with HA.

2 Effective binding parameters

2.1 Effective binding affinities

2.1.1 Monovalent interaction

When the analyte is monovalent, a 1:1 binding model is fit to the data,

$$B = \frac{R_T A}{K_D + A}$$

to produce a $K_D = 124$ μM. In this model, K_D represents the concentration of analyte that produces 50% maximal binding and the concentration of analyte that occupied 50% of bound surface ligands (i.e. $B = R_T/2$ when $A = K_D$).

2.2 Bivalent interaction

We assume that there is a bivalent analyte that is able to bind surface ligand to initially form a monovalent interaction (R_1) followed by a bivalent interaction (R_2). At equilibrium, we have

$$\begin{aligned} K_D^1 R_1 &= RA \\ K_D^2 R_2 &= R_1 A \end{aligned}$$

where A is the concentration of analyte, R is the free surface ligand concentration, and K_D^1 and K_D^2 are the solution and surface dissociation constants, respectively. The equation for conservation of surface ligand is,

$$R_T = R + 2R_1 + 2R_2$$

Using these equations, we can obtain explicit solutions for R, R_1, and R_2 as follows,

$$\begin{aligned} R &= \left[-\left(\frac{K_D^1 K_D^2}{2A} + K_D^2 \right) + \sqrt{\left(\frac{K_D^1 K_D^2}{2A} + K_D^2 \right)^2 + \frac{4R_T K_D^1 K_D^2}{2A}} \right] /2 \\ R_1 &= \frac{AR}{K_D 1} \\ R_2 &= \frac{AR^2}{K_D 1 K_D 2} \end{aligned}$$

As in the monovalent binding example, we can now ask two questions. First, what is the concentration of analyte that produces have maximal binding? To answer this we note that the total amount of analyte bound to the surface is,

$$B = 2R_1 + R_2$$

and the maximal analyte that can bind is R_T (all analyte is bound monovalently). We thus solve for A when $B = R_T/2$ to obtain the concentration of A that produces half-maximal binding (defined as K_D^*),

$$K_D^* = K_D^1/2$$

With the parameters above, we find that in this case $K_{\text{D}}^{*} = 62$ μM. Second, what is the concentration of analyte that occupies 50% of the surface ligands? To answer this question we note that the total concentration of bound surface ligand is,

$$B = 2R_1 + 2R_2$$

and the maximal concentration of bound surface ligand is R_T. We thus solve for A when $B = R_T/2$ to obtain the concentration of A where 50% of the surface ligand is bound (defined as K_{D}^{*}),

$$K_{\text{D}}^{*} = K_{\text{D}}^{1} \frac{K_{\text{D}}^{2}}{2K_{\text{D}}^{2} + R_T} \tag{3}$$

which can be simplified to,

$$K_{\text{D}}^{*} = K_{\text{D}}^{1} \left(\frac{k_{\text{off}}}{2k_{\text{off}} + k_{\text{on}}^{2} R_T} \right) \tag{4}$$

With the fitted values above, we find that in this case $K_{\text{D}}^{*} = 8.22$ μM.

2.3 Effective binding kinetics

2.3.1 Monovalent interaction

Data fitting of the bivalent data (Fig. 3D) revealed that the monovalent off-rate is $k_{\text{off}}^{1} = k_{\text{off}}^{2} = 8.55$ s^{-1}. This large value is consistent with the rapid unbinding observe with monovalent LYVE-1 (Fig. 3C).

2.3.2 Bivalent interaction

In this section we calculate the effective off-rate (k_{off}^{*}) between the bivalent analyte and the monovalent surface ligand when the analyte is bivalently bound to the surface. The calculation is performed in the limit where there is no competition for surface ligand by analyte (i.e. the dilute analyte regime).

The relevant system of equations that determine the probabilities (P) of remaining bound as a function of time are,

$$\begin{aligned} \dot{P}_1 &= -(k_{\text{on}}^{2} R_T + k_{\text{off}}) P_1 + k_{\text{off}} P_2 \\ \dot{P}_2 &= 2k_{\text{on}}^{2} R_T P_1 - 2k_{\text{off}} P_2 \end{aligned} \tag{5}$$

where P_1 represents the monovalent bound state, P_2 the bivalent bound state, k_{off} is the off-rate, k_{on}^{2} is the second-step binding rate, and R_T is the concentration of the surface ligand. The initial conditions are $P_1(t = 0) = 0$ and $P_2(t = 0) = 1$. The lifetime of each state is determined by the probabilities as follows,

$$\tau_1 = \int_0^\infty P_1 dt$$
$$\tau_2 = \int_0^\infty P_2 dt$$

Therefore, the effective off-rate (k^*_{off}) is related to these lifetimes as follows,

$$k^*_{\text{off}} = \frac{1}{2\tau_1 + \tau_2} \tag{6}$$

To determine k^*_{off}, we need to determine τ_1 and τ_2. Taking the integral of equation 7 with respect to time from $t = 0$ to $t = \infty$, we obtain,

$$\begin{aligned} 0 &= -(k^2_{\text{on}} R_T + k_{\text{off}})\tau_1 + k_{\text{off}}\tau_2 \\ -1 &= 2k^2_{\text{on}} R_T \tau_1 - 2k_{\text{off}}\tau_2 \end{aligned} \tag{7}$$

This system can be solved to obtain,

$$\begin{aligned} k^*_{\text{off}} &= \frac{1}{2\tau_1 + \tau_2} \\ &= \frac{2(k_{\text{off}})^2}{3k_{\text{off}} + k^2_{\text{on}} R_T} \end{aligned}$$

Note that this rate depends on the free surface ligand concentration. Using $k_{\text{off}} = 8.55 \text{ s}^{-1}$, $k^2_{\text{on}} = 7.46$ $\mu\text{M}^{-1}\text{s}^{-1}$, and $R_T = 150$ μM (assuming all surface ligand binding sites are free - see subsequent section for details), we have that $k^*_{\text{off}} = 0.128 \text{ s}^{-1}$.

3 Estimating surface concentration of HA

The amount of immobilised HA on the chip surface is 30 RU in Fig. 3C. If one assumes that 30 RU is 0.3 mg/ml (in general 1 mg/ml protein gives 100 RU), then the HA polymer is at $\sim$ 1 μM and the concentration of dimer binding sites is $\sim$ 75 μM (estimating that there are 75 dimer binding sites per HA molecule). Therefore, the total concentration of monomer binding sites is $R_T = 150$ μM.
